# Dual-task gait and gait-cognition coupling in patients with Parkinson’s disease versus healthy older adults: moderation by cognitive status

**DOI:** 10.3389/fnagi.2026.1896308

**Published:** 2026-08-12

**Authors:** Bora Jin, Sang-Myung Cheon

**Affiliations:** Department of Neurology, College of Medicine, Dong-A University, Busan, Republic of Korea

**Keywords:** cognitive-motor associations, dual-task gait, gait analysis, mild cognitive impairment, Parkinson’s disease

## Abstract

**Background:**

Gait impairment and cognitive decline frequently co-occur in Parkinson’s disease (PD), yet how cognitive status modulates dual-task gait-cognition relationships remains poorly characterized.

**Objective:**

To compare spatiotemporal gait parameters and dual-task costs (DTCs) between drug-naïve PD and healthy older adults (HO), and to examine how cognitive status modulates gait-cognition associations within PD.

**Methods:**

One hundred twenty-four participants (65 PD, 59 HO) were prospectively enrolled. PD patients were subclassified into cognitively normal (PDCN, *n* = 38) and mild cognitive impairment (PDMCI, *n* = 27) groups. Gait was assessed using the GAITRite system under four conditions: preferred speed, serial sevens subtraction, cell phone use, and backward walking. Partial Spearman correlations and moderation analyses were conducted.

**Results:**

PD-related gait impairments were more pronounced under dual-task conditions, with cell phone use yielding the greatest between-group differences in spatiotemporal parameters and DTCs. Gait-cognition associations in the total PD group were most consistently observed during cell phone use, involving executive, language, and attention domains. DTCs were specifically correlated with executive and language domains. Subgroup analyses revealed distinct cognitive-motor profiles: PDCN showed a paradoxical pattern in which better visuospatial function was associated with greater gait variability, while PDMCI exhibited broader positive correlations between multiple cognitive domains and gait stability. Moderation analyses confirmed these patterns were statistically distinct.

**Conclusion:**

Dual-task gait assessment using a cell phone paradigm may effectively characterizes cognitive-motor decline in early drug-naïve PD. Cognitive status qualitatively modulates gait-cognition coupling, with domain-specific patterns differing between PDCN and PDMCI, with implications for targeted monitoring and intervention.

## Introduction

Gait is no longer viewed as a purely autonomous motor activity (Yogev-Seligmann et al., 2008). Instead, it is recognized as a complex activity integrated with higher-order cognitive domains, such as executive function, attention, and processing of internal and external stimuli (Amboni et al., 2013). Accordingly, specific gait disturbances have emerged as potential indicators of cognitive deterioration (Verghese et al., 2008). This is particularly relevant in Parkinson’s disease (PD), a neurodegenerative condition wherein both gait and cognition are prominently affected. Gait impairments in PD have been shown to vary according to cognitive subgroup, and patients with mild cognitive impairment (PDMCI) show poorer performance than those with normal cognition (PDCN), a disparity that has been shown to be especially evident under more cognitively demanding conditions (Amboni et al., 2022). Among PDMCI subtypes, multiple-domain and amnestic subtypes have been associated with worse gait patterns, particularly when an additional cognitive load is imposed. Non-amnestic multiple-domain PDMCI is linked to pronounced postural instability and gait disturbances, indicating that the breadth of cognitive involvement may be a key driver of motor deterioration (Goldman et al., 2012).

Beyond its relationship with cognition, gait impairment in PD is among the most common and disabling symptoms, worsening as the disease progresses (Zhang et al., 2024), and has been closely linked to reduced quality of life, increased mortality, and fall risk (Amara et al., 2025; Gonzalez et al., 2022). Furthermore, because walking in daily life frequently involves concurrent activities, dual-task walking has gained considerable attention as a clinically relevant assessment tool. This focus has led to the widespread use of dual-task cost (DTC) metrics (Monaghan et al., 2023). The DTC quantifies the relative change in performance during dual-task gait in comparison with single-task gait, providing valuable insights into the extent of gait deterioration resulting from added cognitive or motor loads. The interaction between gait and cognition is fundamentally based on the concept of limited resource-capacity sharing (Tombu and Jolicoeur, 2003). When a cognitive or motor load is added, the brain must allocate limited neural resources between the two tasks, often leading to cognitive–motor interference, wherein individuals must employ strategies such as task prioritization or compensation to manage the competing demands (Johansson et al., 2021). Prior studies have examined dual-task gait parameters across cognitive subgroups within PD. Di Filippo et al. (2025) demonstrated that dual-task-related gait alterations follow a gradient corresponding to the degree of cognitive dysfunction, becoming progressively more pronounced from PD with normal cognition (PD-noCI) through subjective cognitive impairment (PD-SCI) to MCI (PD-MCI), suggesting that even subclinical cognitive decline may be detectable via dual-task gait. In contrast, a systematic review and meta-analysis by Msigwa et al. (2026) reported that while dual-task gait parameters reliably distinguished PD with cognitive impairment from cognitively normal PD, no significant differences were observed between PD-SCI and PD-noCI, highlighting an unresolved discrepancy regarding the sensitivity of dual-task gait at the earliest stages of cognitive decline. Importantly, both studies lacked a healthy control group, limiting their ability to disentangle disease-specific gait alterations from those attributable to normative aging. Moreover, participants were predominantly levodopa-treated patients across variable disease stages, introducing heterogeneity in baseline gait that complicates interpretation of dual-task effects. Critically, neither study employed dual-task cost (DTC) metrics, which normalize for individual differences in baseline gait performance and thereby provide a more sensitive index of cognitive-motor interference. Furthermore, prior studies have predominantly employed a single cognitive dual-task paradigm, typically serial subtraction or auditory stroop tasks, which may not adequately capture the complexity of real-world cognitive-motor demands. These limitations underscore the need for studies conducted in early-stage, drug-naïve PD cohorts that include a healthy control group, employ DTC as a primary outcome measure, and incorporate ecologically valid dual-task paradigms to better characterize cognitive-motor interactions in PD.

Therefore, the purpose of this study was to compare spatiotemporal gait parameters and DTCs between individuals with PD and HO under single- and dual-task walking conditions, incorporating multiple task conditions of varying ecological validity, with a particular focus on a cell phone use task as an ecologically valid cognitive-motor dual-task reflecting a ubiquitous real-world activity. In addition, we examined the associations between spatiotemporal gait parameters and specific cognitive domains in each group. As a secondary aim, we explored whether these gait–cognition associations differed further in relation to the cognition status within the PD group, comparing patients with PDCN and those with PDMCI. Through this multilevel analytical approach, this study sought to identify the gait metrics and task conditions that were most sensitive to cognitive status and to elucidate the cognitive–motor mechanisms that underlie gait deterioration in PD.

## Methods

### Study population

The participants were prospectively recruited from a tertiary referral hospital between July 2023 and February 2025. The study cohort consisted of HO and patients diagnosed with PD. All patients fulfilled the Movement Disorder Society (MDS) clinical diagnostic criteria for PD and were drug-naïve at the time of the assessment (Postuma et al., 2015). The HO group consisted of caregivers of patients with Parkinsonian disorders and community-dwelling volunteers. For both groups, inclusion was restricted to individuals aged 60–80 years who were independently ambulatory and showed no functional limitations in daily living. Participants in the HO group were required to have no history of neurological deficits or cognitive impairment. All participants underwent global cognitive screening; in addition, patients with PD completed a comprehensive neuropsychological battery. On the basis of the results of this battery, patients with PD were categorized into PDCN and PDMCI in accordance with published diagnostic criteria (Litvan et al., 2012). The exclusion criteria for both groups were as follows: (1) medical or musculoskeletal conditions that could significantly interfere with gait performance; (2) co-existing neurological disorders other than PD; and (3) a history of major psychiatric illness.

### Neuropsychological assessment

All participants underwent global cognitive screening using the Korean version of the Montreal Cognitive Assessment (K-MoCA) (Kang et al., 2009). For the K-MoCA, domain-specific subscores were derived in accordance with a previously proposed categorization (Miyamoto and Miyamoto, 2022), which encompassed visuospatial/executive function, attention, language, memory, and orientation. In addition, all patients with PD underwent a comprehensive neuropsychological battery to evaluate five distinct cognitive domains: (1) attention (Digit Span Forward/Backward and Color Word Stroop Test); (2) language (Boston Naming Test); (3) visuospatial function (Rey-Osterrieth Complex Figure Test copy); (4) memory (Seoul Verbal Learning Test and Rey-Osterrieth Complex Figure Test); and (5) executive function (Controlled Oral Word Association Test) (Yoo et al., 2021). For each test, raw scores were converted into standardized *Z*-scores based on age-, sex-, and education-adjusted normative data. Domain composite scores were calculated by averaging the *Z*-scores of the individual tests within each domain.

### Gait assessment protocol

Gait performance was assessed using the GAITRite Electronic Walkway system (CIR Systems Inc., Peekskill, NY, USA), which consists of a 6-m-long and 0.6-m-wide pressure-sensitive mat embedded with sensors to capture spatiotemporal gait parameters with high resolution. The participants completed all the assessments barefoot. Height, weight, and mean leg length (defined as the distance from the medial malleolus to the anterior inferior iliac spine) were measured before evaluation. Each participant completed the walking trials under the following four conditions: (1) preferred speed, (2) serial sevens subtraction (cognitive dual-task), (3) cell phone use, in which participants typed a pre-specified, randomly ordered numeric sequence using the default numeric keypad on a smartphone while walking (cognitive–motor dual-task), and (4) backward walking (motor-challenging task requiring altered locomotor control). The mean values of the spatiotemporal gait parameters across three trials were used for the analysis. All assessments were conducted in a quiet, well-lit environment, and participants were given standardized instructions prior to each condition to ensure consistency across trials.

The mean values of 16 spatiotemporal gait parameters were collected and categorized into five domains: pace, variability, rhythm, asymmetry, and postural control (Galna et al., 2015). The pace domain included stride velocity (cm/s), step length (cm), and swing time standard deviation (SD) (s). The variability domain consisted of the step time SD (s), stance time SD (s), stride velocity SD (cm/s), and step length SD (cm). The rhythm domain consisted of step time (s), swing time (s), and stance time (s). The asymmetry domain included step-time asymmetry (s), swing-time asymmetry (s), and stance-time asymmetry (s). The postural control domain consisted of step-length asymmetry (cm), base of support (cm), and base of support SD (cm).

DTC was computed for the dual-task conditions (serial sevens subtraction and cell phone use), and an equivalent relative gait cost was calculated for backward walking, using the following formula:

$$\%\Delta =[\frac{\left(\text{Dual}-\text{task condition}-\text{Single}-\text{task condition}\right)}{\text{Single}-\text{task condition}}]\times 100$$

The resulting values were labeled according to the corresponding condition: %Δ2–1 for serial sevens subtraction (cognitive dual-task cost), %Δ3–1 for cell phone use (cognitive–motor dual-task cost), and %Δ4–1 for backward walking (motor-challenge cost), each referenced to the preferred speed condition. The DTC parameter reflected how the additional cognitive and motor loads introduced during the dual-task conditions affected gait performance, thereby integrating both gait and cognitive–motor assessments (Ali et al., 2025). For backward walking, %Δ4–1 is interpreted as the relative gait-performance cost under a motor-challenging condition rather than as evidence of cognitive–motor dual-task interference.

### Ethics approval statement

The protocol of this study was approved by the Ethics Committee of the Dong-A University Hospital. Written informed consent was obtained from all participants. All processes of this study were carried out in accordance with the Declaration of Helsinki.

### Statistical analyses

Continuous variables are presented as mean ± SD, while categorical variables were reported as frequencies. Normality was assessed using the Shapiro–Wilk test. For demographic and clinical characteristics, differences between the HO and total PD groups were examined using the Mann–Whitney U test, chi-square test, or independent *t*-test, as appropriate. For variables requiring covariate adjustment, a ranked analysis of covariance (ANCOVA) was performed, controlling for age, sex, and educational attainment. For comparisons among the three subgroups (HO, PDCN, and PDMCI), the Kruskal–Wallis test, chi-square test, one-way analysis of variance (ANOVA), or ranked ANCOVA with the same covariates was employed as appropriate. For the primary analysis of spatiotemporal gait parameters and DTC, group differences were evaluated using ranked ANCOVA adjusted for age, sex, mean leg length, and educational attainment. The gait profiles were visualized as Z-scores based on the mean and SD of the HO group.

Partial Spearman’s correlation analyses were conducted to examine the relationships between gait and cognition across different levels of comparison. Within the total PD group, correlations were obtained between gait parameters and neuropsychological domain composite scores; this analysis was restricted to parameters that demonstrated significant differences in the primary PD versus HO comparisons, with false discovery rate (FDR) correction applied separately within each task condition. To further explore gait–cognition associations, correlations were also obtained between spatiotemporal gait parameters and total scores and domain-specific subscores of the K-MoCA within each group (PD and HO separately); this analysis was restricted to parameters showing significant PD versus HO group differences, where applicable. As an exploratory subgroup analysis, correlations were also obtained between the spatiotemporal gait parameters and neuropsychological domain composite scores within the PDCN and PDMCI groups separately, restricted to parameters showing significant differences between these two subgroups. For the latter two analyses, nominal *p*-values are reported given their exploratory nature. All the models were adjusted for age, sex, and mean leg length. Although the neuropsychological domain composite scores were derived from age-, sex-, and education-referenced normative data, these covariates were retained to ensure appropriate adjustment of spatiotemporal gait parameters. To formally evaluate whether cognitive status moderated the gait–cognition associations identified in the subgroup correlation analyses, moderation analyses were performed on gait–cognition pairs that showed significant or nominally significant correlations in the PDCN or PDMCI group. Linear regression models were constructed with the cognitive status (PDCN vs. PDMCI) as the moderator, the relevant gait parameter as the predictor, and the corresponding cognitive domain composite score as the outcome. An interaction term (gait parameter × cognitive status) was included to test whether the strength or direction of the association differed significantly between the two subgroups, with all models adjusted for age, sex, educational attainment, and mean leg length. All analyses were conducted using R software (v.4.4.3).

## Results

### Participant characteristics

A total of 124 participants (65 with PD and 59 HO) were enrolled in this study. Within the PD group, 38 patients were categorized as having PDCN and 27 as having PDMCI. In the two-group comparison, patients in the PD group were significantly younger and had lower educational attainment than those in the HO group. However, when comparing the three subgroups (HO, PDCN, and PDMCI), no significant differences were observed in terms of age, sex, mean leg length, or educational attainment. Within the PD group, the disease duration did not differ significantly between the PDCN and PDMCI groups.

In the assessments of global cognitive performance, the total K-MoCA score showed no significant differences between the PD and HO groups. However, a significant difference was observed in the orientation domain, with the PD group scoring lower. In the three-subgroup comparison, both the K-MoCA total score and the score for the orientation domain showed significant differences. The PDMCI group performed significantly worse than the HO group, whereas the PDCN group showed intermediate values that did not reach statistical significance. As expected from the cognitive-based classification, the PDMCI group performed significantly worse than the PDCN group across nearly all neuropsychological indices, with the exception of digit span forward and backward in the attention domain, as detailed in Supplementary Table 1. The demographic and clinical characteristics of the study population are summarized in Table 1.

**Table 1:** Demographics and clinical characteristics of the study participants.

	HO (*N* = 59)	PD (*N* = 65)			*p* value
		All	PDCN (*N* = 38)	PDMCI (*N* = 27)	
Age	72.339 ± 7.519	68.923 ± 5.504	68.842 ± 6.154	69.037 ± 4.544	**0.019** ^**1**^
Sex, M/F	31/28	28/37	18/20	10/17	0.382^2^
Leg length (cm)	85.502 ± 4.482	84.535 ± 4.653	85.322 ± 4.205	83.426 ± 5.093	0.242^3^
Education (yr)	11.627 ± 3.796	10.138 ± 3.539	10.526 ± 3.882	9.593 ± 2.978	**0.030** ^**1**^
Disease duration (m)		17.523 ± 18.870	14.053 ± 13.948	22.407 ± 23.606	0.053^1^
K-MoCA	26.644 ± 1.529	23.776 ± 3.350	25.267 ± 2.815	21.421 ± 2.755^a^	0.098^4^
Visuospatial	3.724 ± 0.523	3.327 ± 0.922	3.667 ± 0.661	2.789 ± 1.032	0.618^4^
Attention	5.672 ± 0.574	5.184 ± 0.858	5.300 ± 0.837	5.000 ± 0.882	0.871^4^
Language	4.793 ± 0.409	4.571 ± 0.913	4.767 ± 0.504	4.263 ± 1.284	0.074^4^
Executive	3.397 ± 0.591	2.673 ± 1.162	3.000 ± 0.983	2.158 ± 1.259	0.675^4^
Memory	3.052 ± 1.099	1.796 ± 1.399	2.400 ± 1.276	0.842 ± 1.015	0.135^4^
Orientation	5.983 ± 0.131	5.939 ± 0.242	5.933 ± 0.254	5.947 ± 0.229^a^	**0.005** ^**4**^

Values are presented as mean ± standard deviation for continuous variables and *n* for categorical variables. Normality was assessed using the Shapiro–Wilk test. Group differences between the HO and PD total groups were tested using Mann–Whitney ^1^U test, ^2^chi-square test, ^3^independent t-test, or ^4^ranked ANCOVA (adjusted for age, sex, and educational attainment). For comparisons among the three subgroups (HO, PDCN, and PDMCI), the Kruskal–Wallis test, chi-square test, ANOVA, or ranked ANCOVA (adjusted for age, sex, and educational attainment) were employed. Superscripts denote the statistical tests used. Bold values indicate statistically significant differences (*p* < 0.05) between the HO and PD total groups. ^a^Significant difference compared with the HO group. ^b^Significant difference between the PDCN and PDMCI groups.

HO, Healthy Older adults; K-MoCA, Korean-Montreal Cognitive Assessment; PD, Parkinson’s disease; PDCN, Parkinson’s disease with Cognitively Normal; PDMCI, Parkinson’s disease with Mild Cognitive Impairment.

### Spatiotemporal gait parameters and dual-task cost

Across most gait parameters, the PD group demonstrated worse performance than the HO group, and this pattern became more pronounced under dual-task conditions. Specifically, under the cell phone use and backward-walking conditions, a greater number of parameters showed statistically significant group differences in comparison with the preferred speed condition, particularly within the pace and asymmetry domains. In contrast, the serial sevens subtraction condition yielded fewer significant between-group differences. Notably, certain parameters within the variability and postural control domains were worse in the HO group.

**Figure 1:**
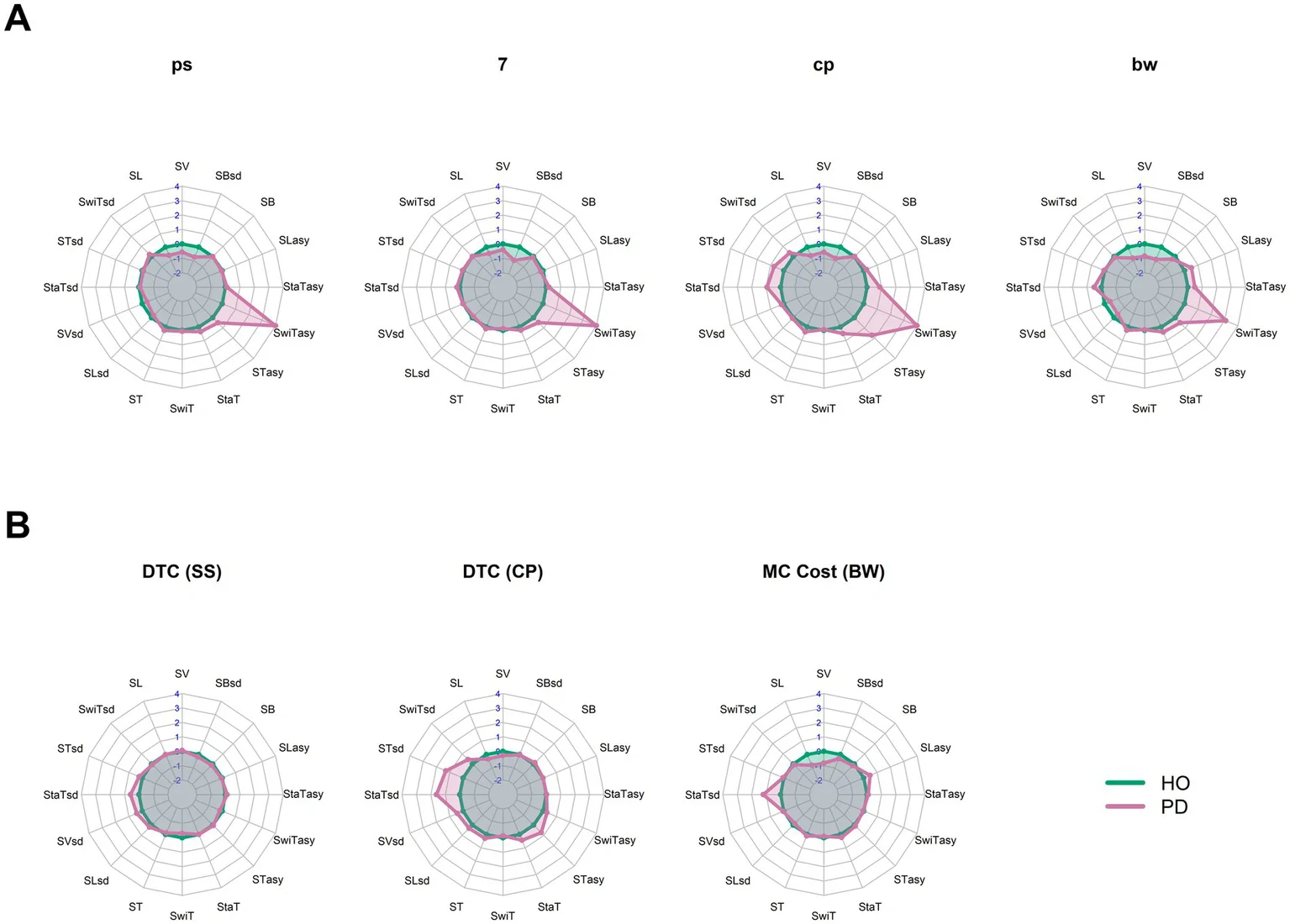
Comparison of gait profiles among healthy older adults and patients with PD. **(A)** Standardized spatiotemporal gait parameters under four walking conditions: preferred speed, serial sevens subtraction, cell phone use, and backward walking. **(B)** Comparison of relative gait costs calculated for each task condition, including dual-task costs for serial sevens subtraction and cell phone use, and motor-challenging cost for backward walking. All parameters are standardized based on the mean and standard deviation of the healthy older adults group (*z*-score = 0). Values further from the center indicate greater deviation or impairment compared to the healthy baseline. Notably, certain parameters in the PD group (e.g., swing time asymmetry) that exceeded the maximum visualization scale (*z*-score > 4) were clipped at the outer boundary to maintain comparative readability across all gait domains. Asy, asymmetry; bw, backward walking; cp, cell phone use; DTC, dual-task cost; HO, healthy older adults; MC cost, motor-challenging cost; PD, Parkinson’s disease; ps, preferred speed; SB, support base; sd, standard deviation; StaT, stance time; SL, step length; ST, step time; SV, stride velocity; SwiT, swing time; 7, serial sevens subtraction.

A similar pattern was observed for DTC. In comparison with the serial sevens subtraction condition (%Δ2–1), both the cell phone use (%Δ3–1) and backward-walking (%Δ4–1) conditions were associated with a greater number of parameters showing significant between-group differences. Furthermore, the directionality of the DTC, which reflected greater cognitive–motor interference in the PD group, was more consistently observed in the cell phone use condition. Overall, the cell phone use condition emerged as the most sensitive task, showing the greatest number of significant between-group differences across both spatiotemporal gait parameters and DTCs, as further illustrated in Figure 1. The spatiotemporal gait parameters and dual-task costs across the conditions are summarized in Tables 2, 3, respectively.

**Table 2:** Spatiotemporal gait parameters across task conditions between healthy older adults and patients with Parkinson’s disease.

	PS			Serial 7 s			CP			BW		
	HO	PD	*p_FDR_*	HO	PD	*p_FDR_*	HO	PD	*p_FDR_*	HO	PD	*p_FDR_*
Pace												
SV	113.129 ± 22.649	100.110 ± 25.171	**0.010**	99.926 ± 26.966	89.176 ± 26.585	0.130	82.579 ± 24.108	68.943 ± 22.937	**0.003**	76.465 ± 22.799	57.128 ± 20.556	**<0.001**
SL	59.514 ± 9.784	53.598 ± 10.107	**0.002**	54.606 ± 11.477	49.213 ± 10.891	0.064	48.389 ± 10.353	41.758 ± 10.129	**<0.001**	39.428 ± 10.872	30.544 ± 10.029	**<0.001**
SwiT sd	0.021 ± 0.009	0.023 ± 0.015	0.781	0.027 ± 0.019	0.027 ± 0.018	0.862	0.042 ± 0.034	0.054 ± 0.046	**0.018**	0.058 ± 0.084	0.051 ± 0.037	0.435
Variability												
ST sd	0.034 ± 0.015	0.033 ± 0.027	0.112	0.039 ± 0.039	0.040 ± 0.054	0.862	0.056 ± 0.045	0.088 ± 0.098	**0.016**	0.057 ± 0.061	0.060 ± 0.039	0.084
StaT sd	0.044 ± 0.023	0.043 ± 0.048	0.089	0.048 ± 0.062	0.060 ± 0.107	0.862	0.069 ± 0.057	0.123 ± 0.155	**0.016**	0.059 ± 0.029	0.074 ± 0.044	0.060
SV sd	7.908 ± 3.417	6.691 ± 3.160	**0.028**	6.929 ± 2.523	6.936 ± 2.827	0.895	7.944 ± 2.991	8.435 ± 3.048	0.341	10.281 ± 3.288	8.940 ± 3.290	**0.036**
SL sd	3.492 ± 1.627	3.175 ± 1.405	0.144	3.550 ± 1.430	3.380 ± 1.185	0.862	4.330 ± 2.042	4.456 ± 1.618	0.338	6.569 ± 2.953	5.524 ± 1.834	**0.031**
Rhythm												
ST	0.541 ± 0.055	0.554 ± 0.070	0.711	0.569 ± 0.083	0.579 ± 0.106	0.934	0.611 ± 0.093	0.645 ± 0.119	0.169	0.538 ± 0.089	0.559 ± 0.107	0.511
SwiT	0.403 ± 0.035	0.405 ± 0.039	0.867	0.413 ± 0.050	0.407 ± 0.057	0.548	0.430 ± 0.054	0.426 ± 0.061	0.851	0.370 ± 0.068	0.366 ± 0.069	0.762
StaT	0.672 ± 0.079	0.699 ± 0.107	0.495	0.720 ± 0.130	0.750 ± 0.172	0.862	0.791 ± 0.148	0.863 ± 0.206	0.076	0.705 ± 0.119	0.750 ± 0.157	0.271
Asymmetry												
ST asy	0.018 ± 0.016	0.026 ± 0.025	0.495	0.020 ± 0.020	0.030 ± 0.031	0.130	0.019 ± 0.018	0.050 ± 0.045	**<0.001**	0.024 ± 0.023	0.035 ± 0.028	**0.035**
SwiT asy	0.013 ± 0.009	0.091 ± 0.154	**0.015**	0.013 ± 0.011	0.093 ± 0.159	**0.017**	0.018 ± 0.017	0.106 ± 0.154	**<0.001**	0.019 ± 0.023	0.090 ± 0.129	**<0.001**
StaT asy	0.017 ± 0.016	0.019 ± 0.015	0.711	0.016 ± 0.014	0.018 ± 0.016	0.862	0.018 ± 0.017	0.033 ± 0.030	**0.006**	0.021 ± 0.016	0.029 ± 0.022	0.057
Postural control												
SL asy	2.369 ± 1.650	2.300 ± 1.813	0.781	2.687 ± 1.843	2.395 ± 2.042	0.548	2.375 ± 1.789	2.733 ± 2.692	0.722	3.260 ± 3.010	4.804 ± 3.325	**0.011**
SB	9.722 ± 2.569	9.717 ± 2.617	0.867	9.986 ± 2.848	9.761 ± 2.734	0.880	10.025 ± 3.239	10.089 ± 2.541	0.615	16.898 ± 4.071	15.876 ± 3.450	0.123
SB sd	2.103 ± 0.711	1.583 ± 0.501	**<0.001**	2.059 ± 0.578	1.487 ± 0.512	**<0.001**	2.649 ± 0.725	2.037 ± 0.797	**<0.001**	3.426 ± 1.380	2.166 ± 0.867	**<0.001**

Comparison of gait parameters between PD and healthy older adult groups across four task conditions: preferred speed, dual task-7, dual task-cell phone, and backward walking. Values are presented as means ± standard deviation, and group differences were tested using ranked ANCOVA adjusted for age, sex, leg length, and educational attainment. *p*-values were adjusted for multiple comparisons using the false discovery rate (FDR) method, and significant differences (FDR-adjusted *p* < 0.05) are indicated in bold.

Asy, asymmetry; BW, Backward walking; CP, Cell phone use; PS, Preferred Speed; SB, Support Base; sd, standard deviation; StaT, Stance Time; SL, Step Length; ST, Step Time; SV, Stride Velocity; SwiT, Swing Time.

**Table 3:** Relative gait costs across task conditions between healthy older adults and patients with Parkinson’s disease.

	%Δ2–1			%Δ3–1			%Δ4–1		
	HO	PD	*p_FDR_*	HO	PD	*p_FDR_*	HO	PD	*p_FDR_*
Pace									
SV	−12.413 ± 11.995	−11.648 ± 11.353	0.872	−27.835 ± 11.558	−31.135 ± 15.456	0.409	−33.224 ± 12.262	−43.303 ± 12.580	**<0.001**
SL	−8.693 ± 8.867	−8.682 ± 7.995	0.911	−19.034 ± 9.745	−22.270 ± 12.079	0.085	−34.489 ± 11.361	−43.535 ± 12.605	**<0.001**
SwiT sd	35.762 ± 75.464	30.975 ± 75.527	0.872	103.966 ± 144.286	166.845 ± 181.011	**0.030**	225.443 ± 547.553	178.905 ± 282.410	0.603
Variability									
ST sd	14.559 ± 72.780	32.186 ± 156.132	0.748	64.949 ± 106.403	202.125 ± 287.174	**0.002**	105.759 ± 333.161	123.215 ± 180.463	**0.011**
StaT sd	9.278 ± 81.221	56.765 ± 228.759	0.265	62.343 ± 108.813	238.596 ± 345.141	**<0.001**	52.649 ± 79.858	148.855 ± 216.140	**0.011**
SV sd	−0.693 ± 48.585	19.152 ± 67.260	0.497	15.345 ± 68.351	44.696 ± 69.641	**0.007**	47.939 ± 66.460	51.050 ± 73.658	0.903
SL sd	10.423 ± 43.617	19.619 ± 55.761	0.748	35.651 ± 65.419	56.527 ± 70.232	0.059	115.190 ± 122.601	97.766 ± 96.404	0.671
Rhythm									
ST	5.003 ± 8.377	4.082 ± 10.211	0.812	12.877 ± 11.728	16.329 ± 16.606	0.502	−0.474 ± 12.017	0.797 ± 14.312	0.806
SwiT	2.481 ± 7.270	0.233 ± 7.954	0.265	6.770 ± 10.546	5.329 ± 11.690	0.819	−8.325 ± 13.785	−9.720 ± 13.590	0.712
StaT	6.752 ± 10.828	6.740 ± 13.502	0.912	17.352 ± 14.254	23.509 ± 23.335	0.417	4.834 ± 12.050	7.485 ± 16.200	0.458
Asymmetry									
ST asy	108.850 ± 362.762	124.567 ± 356.476	0.839	88.427 ± 213.026	246.525 ± 401.388	**0.004**	238.068 ± 568.087	293.374 ± 842.448	0.811
SwiT asy	121.638 ± 395.234	54.802 ± 208.359	0.839	192.374 ± 467.188	332.675 ± 1216.408	0.417	279.263 ± 703.600	246.352 ± 627.906	0.903
StaT asy	50.381 ± 167.027	69.419 ± 260.892	0.748	158.093 ± 455.487	168.029 ± 260.873	0.174	242.799 ± 599.225	289.027 ± 615.571	0.335
Postural control									
SL asy	136.125 ± 472.668	110.701 ± 454.412	0.748	122.246 ± 400.241	121.915 ± 342.742	0.544	182.593 ± 441.999	390.656 ± 790.076	**0.022**
SB	2.695 ± 15.629	0.860 ± 12.627	0.839	3.269 ± 21.242	5.772 ± 16.419	0.417	78.946 ± 41.992	72.293 ± 51.208	0.335
SB sd	4.909 ± 32.339	−0.561 ± 37.560	0.748	38.922 ± 54.783	37.577 ± 66.256	0.544	72.341 ± 64.915	52.640 ± 89.435	**0.022**

***%*** $\Delta :\frac{\text{dual task}-\text{preferred speed}}{\text{preferred speed}}\times 100$ .

Dual task cost (DTC) was calculated as [(dual task - single task) / single task] × 100 (%). %Δ2–1 = serial 7 s subtraction; %Δ3–1 = cell phone use; %Δ4–1 = backward walking, each relative to single task. Values are presented as means ± standard deviation. Group differences were tested using ranked ANCOVA adjusted for age, sex, leg length and educational attainment. FDR-adjusted *p*-values are presented and significant values (*p* < 0.05) are shown in bold.

Asy, asymmetry; BW, Backward walking; CP, Cell phone use; PS, Preferred Speed; SB, Support Base; sd, standard deviation; StaT, Stance Time; SL, Step Length; ST, Step Time; SV, Stride Velocity; SwiT, Swing Time.

### Partial correlation between gait and cognitive domains

Partial correlation analyses within the entire PD group revealed that both spatiotemporal gait parameters and DTCs were significantly associated with the five cognitive domains derived from the neuropsychological battery. Reflecting the prominent group differences observed in Tables 2, 3, these associations were most extensive during the cell phone use task, which demonstrated a consistently dominant correlation pattern across multiple domains after FDR correction. This finding was not merely attributable to the larger number of parameters included in this condition. Performance in the preferred speed condition was primarily linked to the attention domain. In contrast, gait during the cell phone use task showed a broader pattern of associations, correlating significantly with the attention, executive, and language domains. Under these conditions, pace-related parameters, including stride velocity and step length, were positively correlated with cognitive scores across multiple domains. Conversely, increased stance time, step time SD, step time, and swing-time asymmetry were negatively correlated with cognitive scores. Notably, swing-time asymmetry was the only gait parameter that was significantly correlated with the memory domain, and this association was observed across both the preferred speed and cell phone use conditions.

Regarding DTC, the correlation patterns showed a more concentrated distribution, primarily within the executive and language domains. The DTC for step length showed significant positive correlations with both executive and language domains. Furthermore, the higher costs associated with step time SD and stance time SD were consistently linked to lower scores in the executive and language domains. The correlation results are presented in Table 4.

**Table 4:** Partial Spearman correlations between spatiotemporal gait parameters, dual-task costs, and cognitive domain scores in patients with Parkinson’s disease.

	r	*p*_fdr_
Spatiotemporal gait parameters		
SV_ps – attention domain	0.336	0.045
SL_ps – attention domain	0.342	0.045
SwiTasy_ps – attention domain	−0.329	0.045
SwiTasy_ps – memory domain	−0.337	0.045
SwiTasy_ps – visuospatial domain	−0.379	0.045
SV_cp – executive domain	0.409	0.048
SV_cp – language domain	0.343	0.049
SV_cp – attention domain	0.337	0.049
SL_cp – executive domain	0.335	0.049
SL_cp – language domain	0.370	0.049
SL_cp – attention domain	0.325	0.049
STsd_cp – executive domain	−0.326	0.049
StaT_cp – executive domain	−0.355	0.049
STasy_cp – attention domain	−0.340	0.049
SwiTasy_cp – memory domain	−0.336	0.049
SV_bw – attention domain	0.408	0.040
Dual-task cost parameters		
SL_DTCcp - executive domain	0.391	0.017
SL_DTCcp - language domain	0.356	0.027
StaTsd_DTCcp - executive domain	−0.431	0.014
StaTsd_DTCcp - language domain	−0.364	0.027
STsd_DTCcp - executive domain	−0.409	0.015
STsd_DTCcp - language domain	−0.350	0.027

Partial Spearman correlations were performed between gait parameters showing significant group differences and neuropsychological test scores in PD patients (*n* = 65), controlling for age, sex, and leg length. *p*-values were corrected using the FDR method within each condition separately. Only significant correlations (FDR-adjusted *p* < 0.05) are presented.

Asy, asymmetry; cp, cell phone use; DTC, dual-task cost; sd, standard deviation; StaT, Stance Time; SL, Step Length; ST, Step Time; SV, Stride Velocity; SwiT, Swing Time.

**Figure 2:**
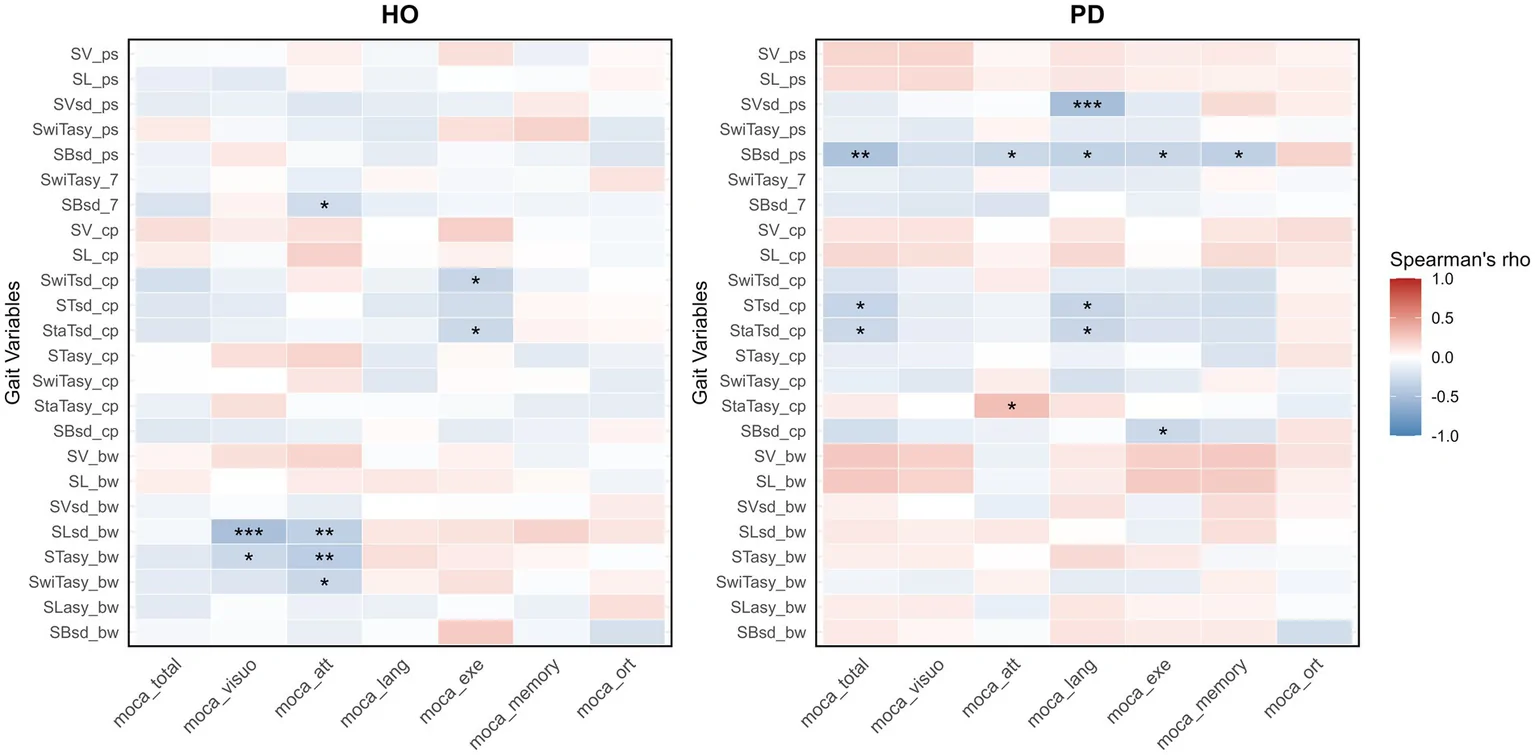
Partial correlation heatmap between cognitive domains and spatiotemporal gait parameters across task conditions in healthy older adults and patients with PD. This heatmap illustrates the partial correlation coefficients between significant gait parameters and cognitive domains from K-MoCA for the healthy older adults and Parkinson’s disease groups. Color intensity reflects the magnitude of Spearman’s *rho*, where red indicates a positive correlation and blue indicates a negative correlation. Grey cells indicate instances where correlation coefficients could not be calculated due to zero variance (e.g., ceiling effects in cognitive scores). Individual tiles marked with a black asterisk (*) represent statistically significant associations at a nominal *p*-value < 0.05 (*p < 0.05, **p < 0.01, and ***p < 0.001). All analyses were adjusted for age, sex, leg length, and educational attainment to account for potential confounding factors. asy, asymmetry; BW, backward walking; CP, cell phone use; PD, Parkinson’s disease; PS, preferred speed; SB, base of support; SD, standard deviation; StaT, stance time; SL, step length; ST, step time; SV, stride velocity; SwiT, swing time; moca_visuo, visuospatial function; moca_att, attention; moca_lang, language; moca_exe, executive function; moca_ort, orientation; 7, serial sevens subtraction.

Beyond the within-PD analyses, gait–cognition associations were examined in both the PD and HO groups using the domain-specific subscores of the K-MoCA to provide a broader comparative perspective. In the PD group, significant associations were observed across multiple gait parameters, predominantly within the language, attention, and executive domains, and were most evident during the preferred speed and cell phone use conditions. In the HO group, significant gait–cognition pairs were less frequent overall but were nonetheless present, with associations most evident in the attention, visuospatial, and executive domains, particularly during cell phone use and backward-walking conditions. These correlation patterns are shown in Figure 2.

**Figure 3:**
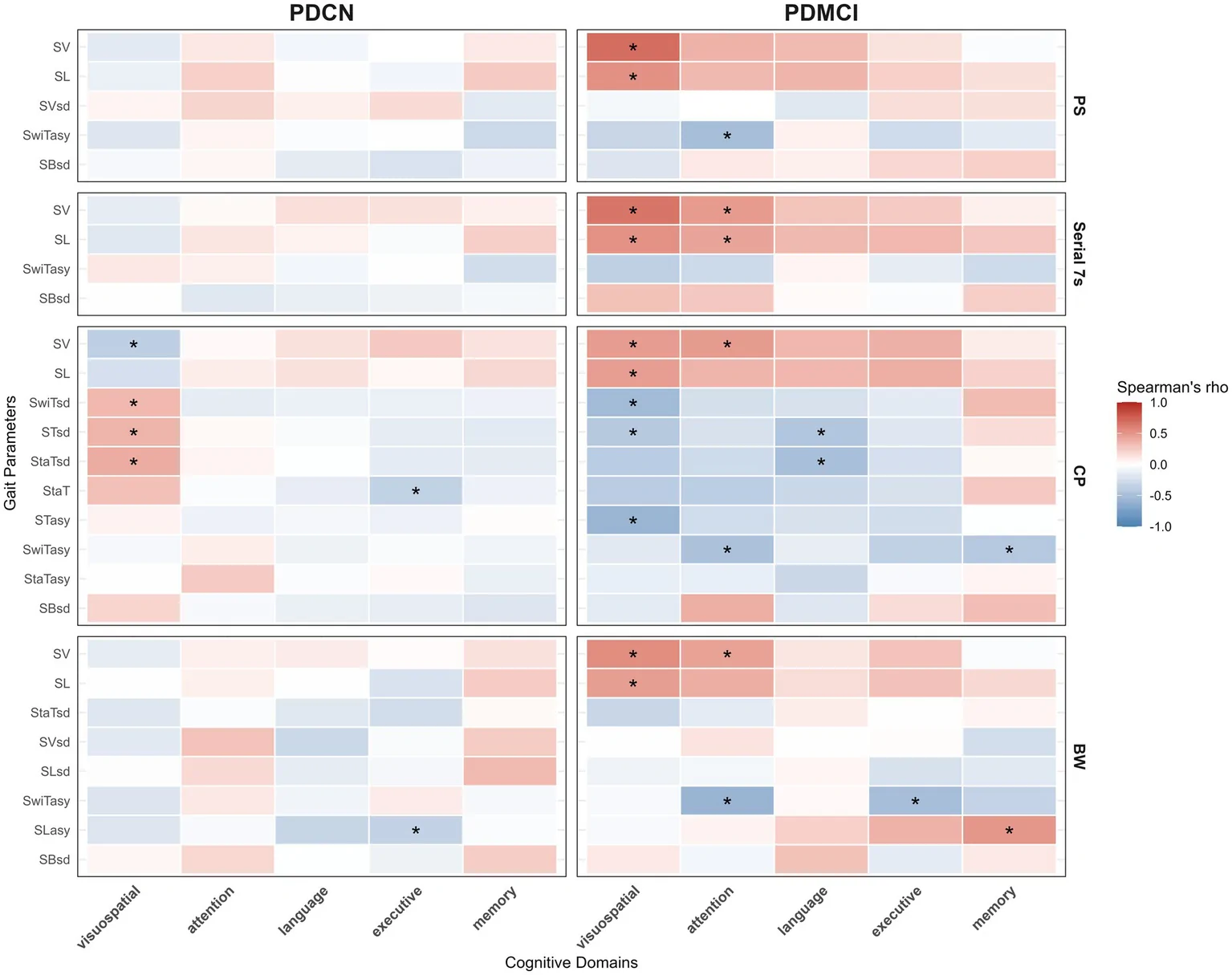
Partial correlation heatmap between cognitive domains and spatiotemporal gait parameters across task conditions in PD patients with cognitively normal and mild cognitive impairment. This heatmap illustrates the partial correlation coefficients between five cognitive domain scores and significant gait parameters, stratified by cognitive status (cognitively normal and mild cognitive impairment) and four distinct walking task conditions (preferred speed, Serial 7 s subtraction, cell phone use, and backward walking). Color intensity reflects the magnitude of Spearman’s rho, where red indicates a positive correlation and blue indicates a negative correlation. Individual tiles marked with a black asterisk (*) represent statistically significant associations at a nominal *p*-value < 0.05. All analyses were adjusted for age, sex, and mean leg length to account for potential confounding factors. asy, asymmetry; BW, backward walking; CP, cell phone use; PDCN, Parkinson’s disease with cognitively normal; PDMCI, Parkinson’s disease with mild cognitive impairment; PS, preferred speed; SB, support base; sd, standard deviation; StaT, stance time; SL, step length; ST, step time; SV, stride velocity; SwiT, swing time; 7, Serial 7 s subtraction.

As a secondary exploratory analysis, to examine whether cognitive status further modulated gait–cognition associations within the PD group, partial correlations were conducted separately for the PDCN and PDMCI groups using spatiotemporal gait parameters that showed significant differences between the two subgroups. These analyses revealed distinct cognitive–motor profiles, with the specific domains and directionality of associations differing markedly between the two groups. In the PDCN group, significant associations were sparse overall and were primarily concentrated within the visuospatial domain during the cell phone use task. Notably, better visuospatial performance was paradoxically associated with both increased gait variability (higher step time SD and stance time SD) and reduced stride velocity. In contrast, the executive domain in the PDCN group showed a more expected relationship, where better executive function was linked to improved gait performance, as reflected by the reduced stance time and step-length asymmetry. The PDMCI group showed a more extensive and widespread correlation pattern, wherein better performance across multiple cognitive domains, particularly the attention and visuospatial domains, was consistently associated with better gait stability. These subgroup-specific correlation patterns are shown in Figure 3 (nominal *p* < 0.05).

To determine whether the specific correlation patterns observed in the heatmaps were statistically driven by cognitive status rather than chance, a formal moderation analysis was performed on the previously identified significant pairs. This analysis aimed to verify whether the strength or direction of the gait–cognition associations were significantly different between the PDCN and PDMCI groups.

Moderation analysis confirmed that cognitive status significantly moderated several of these relationships even after adjusting for age, sex, educational attainment, and mean leg length. Specifically, the paradoxical associations observed in the PDCN group involving the visuospatial domain were found to be statistically distinct from the patterns observed in the PDMCI group (*p* < 0.05 for interaction terms), supporting the notion that the gait–cognition coupling pattern is fundamentally altered in relation to the cognitive status. The moderation effects and associated interaction terms are summarized in Table 5.

**Table 5:** Moderation effects of cognitive status on the relationship between cognitive domains and gait parameters.

Gait parameter	Cognitive domain	Task	Interaction β (SE)	*p* value
Pace				
SV	Visuospatial	Preferred speed	16.00 (6.38)	0.015
	Visuospatial	Serial 7 s subtractions	19.20 (6.65)	0.006
	Visuospatial	Cell phone use	16.20 (5.79)	0.008
SL	Visuospatial	Serial 7 s subtractions	5.80 (2.67)	0.034
	Visuospatial	Cell phone use	5.28 (2.43)	0.034
SwiTsd	Visuospatial	Cell phone use	−0.03 (0.01)	0.040
Variability				
STsd	Language	Cell phone use	−0.06 (0.02)	0.026
	Visuospatial	Cell phone use	−0.05 (0.03)	0.049
StaTsd	Language	Cell phone use	−0.09 (0.04)	0.016
Asymmetry				
STasy	Visuospatial	Cell phone use	−0.03 (0.01)	0.009
SwiTasy	Executive	Backward walking	−0.13 (0.05)	0.014
Postural control				
SLasy	Executive	Backward walking	3.15 (1.30)	0.018

Only significant interactions (*p* < 0.05) are presented. β represents the interaction term (Cognitive Domain × Group), indicating the difference in slopes between PDCN and PDMCI (Reference: PDCN). All models were adjusted for age, sex, educational attainment, and mean leg length.

asy, asymmetry; sd, standard deviation; StaT, Stance Time; SL, Step Length; ST, Step Time; SV, Stride Velocity; SwiT, Swing Time.

## Discussion

This study examined spatiotemporal gait performance and its relationship with cognitive function in individuals with early-stage PD and HO, with the aim of characterizing not only group-level differences in gait, but also how gait–cognition associations differed between the two groups and within the PD group in relation to cognitive status. Gait impairments in PD were more pronounced under dual-task conditions, with the cell phone use task emerging as the most sensitive condition in this sample. Gait–cognition associations within the PD group were most extensive during this condition, predominantly involving the executive, language, and attention domains. Furthermore, distinct cognitive–motor profiles were observed between PDCN and PDMCI, suggesting that cognitive status may qualitatively modulate the nature of gait–cognition interactions in PD.

### Dual-task conditions and cognitive–motor interference

In comparison with single-task walking, dual-task conditions revealed more pronounced between-group differences, particularly within the pace, variability, and asymmetry domains, and most consistently in the cell phone use task. This is consistent with the concept of limited resource-capacity sharing (Tombu and Jolicoeur, 2003), in the addition of a secondary cognitive or motor task competes for the same neural resources required for locomotion. In healthy individuals, walking is largely automatized and requires minimal attentional resources (Clark, 2015). However, in PD, the degeneration of basal ganglia circuitry disrupts the automaticity of gait, increasing its dependence on cortical attentional networks (Kelly et al., 2012). Consequently, when a concurrent task is introduced, individuals with PD are less able to sustain gait performance, causing disproportionate deterioration in comparison with HO (Kusleikiene et al., 2025).

Visualization of spatiotemporal gait parameters and DTCs as Z-scores referenced to the HO group revealed that the swing-time asymmetry showed the largest deviation between the groups. Notably, the swing-time asymmetry already differed significantly between the groups under the preferred speed condition; as a result, the additional increment attributable to dual-task interference, as reflected in the DTC, was comparatively modest. In contrast, the variability domain parameters showed more prominent differences in DTC, suggesting that gait variability is more susceptible to cognitive–motor loading than asymmetry. As previously demonstrated in healthy populations, gait variability is considered a sensitive indicator of attentional resources engaged in motor control, with elevated variability reflecting a shift away from automatic locomotor processing toward more deliberate, attention-demanding control (Riedel et al., 2024). In this context, the disproportionate increase in gait variability observed in patients with PD under dual-task conditions may reflect a greater reliance on attentional resources to maintain locomotion, underscoring its clinical relevance as a marker of cognitive–motor vulnerability. Taken together, even after adjusting for age, sex, and leg length, individuals with early-stage PD demonstrated disease-specific gait alterations in comparison with HO, and these differences were further amplified when concurrent cognitive and motor demands were imposed.

### Cell phone use as the most sensitive dual-task condition

Among the four walking conditions examined, the cell phone use task consistently produced the greatest number of significant between-group differences in the spatiotemporal gait parameters and DTCs. Cell phone use has become an inseparable part of contemporary life and is frequently employed during locomotion in everyday settings, such as commuting and transit. Previous studies have demonstrated that cell phone use during walking increases DTCs across multiple spatiotemporal gait parameters, even in children and young adults (Lee and Shin, 2024), underscoring the substantial cognitive–motor demands imposed by this activity. Unlike the serial sevens subtraction task, which is a purely cognitive secondary task with variable performance across individuals, the cell phone use task simultaneously imposes cognitive and motor demands, requiring visual attention, manual dexterity, and cognitive processing. This multicomponent nature, combined with the ecological validity of cell phone use as a ubiquitous daily-life activity, may render this task particularly sensitive to the limited resource capacity of individuals with PD. This is consistent with previous studies demonstrating that cell phone use during walking produces the most pronounced gait impairments among daily-life dual tasks in patients with PD (Yamada et al., 2020). This pattern is further supported by evidence from healthy older adults showing that combined cognitive–motor dual-task conditions produce disproportionately greater gait impairments than either cognitive or motor tasks alone (Nedović et al., 2024; Nedović et al., 2025). These observations suggest that the cell phone use condition may serve as a clinically useful dual-task paradigm in the assessment of cognitive–motor function in PD, though replication in larger and more diverse cohorts is warranted.

### Paradoxical findings in variability and postural control domains

Interestingly, certain parameters within the variability and postural control domains were paradoxically higher in the HO group than in the PD group. This unexpected finding warrants careful interpretation. The participants with PD in the present study were significantly younger than those in the HO group and were in the early stages of the disease, with minimal postural instability. Postural instability and increased gait variability in PD tend to emerge and worsen with disease progression, indicating that these deficits may not yet fully manifest in early-stage patients. Furthermore, since age is a potent and independent determinant of gait variability (Mukli et al., 2025), the significant age difference between the two groups likely exerted a dominant influence on these specific metrics. Although age, sex, and leg length were included as covariates in the statistical models, the residual influence of age-related physiological changes in the HO group may not have been eliminated. Thus, in the early stages of PD, the influence of normal aging on gait variability and postural control can be more pronounced than that of disease-specific alterations. Collectively, these findings highlight that gait in patients with early PD reflects a complex interplay of disease-related and demographic factors, underscoring the need to interpret between-group differences within the context of the underlying cohort characteristics.

### Gait–cognition associations and cognitive domain specificity

Within the total PD group, partial correlation analyses revealed significant associations between gait parameters and neuropsychological domains, most prominently during the cell phone use task, in which significant correlations were observed with the executive, language, and attention domains. The predominance of attention and executive function in gait–cognition associations is consistent with a substantial body of literature implicating prefrontal circuits in the regulation of dual-task gait (Clark, 2015; Ohsugi et al., 2013; Doi et al., 2013). Prefrontal circuits are known to be functionally compromised in patients with PD, partly as a consequence of disrupted basal ganglia-cortical connectivity (Hjelle et al., 2025), and their dysfunction may underlie both the attentional deficits and gait deterioration observed in this population. The involvement of the language domain is another notable finding, since language processing shares neural substrates with executive function and working memory (De Baene et al., 2015), and this finding may reflect the broader cognitive demands imposed by the cell phone use task. Regarding DTC, correlations were concentrated within the executive and language domains, suggesting that the ability to maintain gait performance under dual-task conditions is particularly sensitive to frontal lobe integrity in patients with PD.

Importantly, the pattern of gait–cognition associations differed between the PD and HO groups when examined using domain-specific subscores derived from the K-MoCA. While the PD group demonstrated associations spanning multiple cognitive domains, the HO group showed a more limited and circumscribed pattern of correlations confined to the attention, visuospatial, and executive domains during the cell phone use and backward-walking conditions. This observation suggests that the coupling between gait and cognition is not exclusive to pathological conditions, but is also present in healthy aging, albeit in a more limited fashion. The broader and more extensive gait–cognition associations observed in PD likely reflect the amplified dependence on cognitive resources for locomotor control that accompanies neurodegeneration.

### Subgroup differences: exploratory cognitive–motor profiles between PDCN and PDMCI

In the subgroup analysis, the PDMCI group, as anticipated, exhibited more extensive gait–cognition associations than the PDCN group, and the specific domains and directionality of these associations differed markedly between the two groups, suggesting that cognitive status may qualitatively modulate the nature of cognitive–motor interactions in PD, though these findings should be interpreted with caution given the exploratory nature of the subgroup analysis and the modest sample sizes involved.

Of particular interest was the pattern observed in the PDCN group during the cell phone use task. Better visuospatial function is notably associated with increased gait variability. While seemingly counterintuitive, this divergent pattern may suggest a potential shift in resource allocation, possibly reminiscent of a posture-second strategy in which visuospatial resources are preferentially directed toward the cell phone use task at the expense of gait stability (Yogev-Seligmann et al., 2012). Notably, this pattern contrasts with prior studies reporting posture-second strategies primarily in individuals with MCI (Johansson et al., 2021) and may reflect the fact that such a flexible, though potentially destabilizing, re-allocation of resources requires sufficient cognitive reserve (Huxhold et al., 2006). Conversely, stronger executive function in the PDCN group was associated with improved gait stability, a finding consistent with prior evidence implicating the prefrontal cortex in both executive cognitive control and the regulation of locomotion under dual-task conditions (Holtzer et al., 2011; Patel et al., 2014). Thus, preserved executive resources may facilitate gait coordination even in the presence of competing task demands. Therefore, the dissociation between visuospatial and executive correlates in PDCN suggests that these two cognitive domains may relate to locomotor control in qualitatively different ways during cognitively demanding tasks, a finding that remains preliminary and warrants further investigation into the domain-specific mechanisms underlying cognitive–motor interactions in early PD.

In contrast, the PDMCI group showed a broader and more consistent pattern of positive correlations between gait stability and performance across multiple cognitive domains. This may indicate that locomotor control had become increasingly dependent on a wider range of cognitive resources, possibly reflecting a more vulnerable cognitive–motor system in which gait stability is more closely tied to overall cognitive capacity. While these findings could be interpreted in the context of task prioritization, direct evidence for such strategies would require the concurrent measurement of secondary task performance. Although the absence of cognitive cost measurements limited our ability to definitively conclude a specific postural prioritization strategy, the addition of a formal moderation analysis provided preliminary statistical support for these exploratory findings. The significant interaction effects suggested that the paradoxical association between visuospatial function and several gait parameters in the PDCN group may represent a statistically distinct pattern from that of the PDMCI group. Taken together, these results suggest that cognitive status may meaningfully modulate the gait–cognition coupling pattern, potentially reflecting a shift in locomotor control strategies as cognitive impairment emerges in PD. Replication in larger, well-characterized cohorts is nonetheless needed before firm conclusions can be drawn.

The present study has several limitations that require consideration. First, the cross-sectional design precluded causal inferences regarding the directionality of the gait–cognition associations. Second, the PD group represented patients at an early stage of the disease, which may limit the generalizability of the findings to patients with advanced PD. Third, despite covariate adjustment, the significant age difference between the PD and HO groups may have influenced some of the observed patterns, particularly within the variability and postural control domains. Fourth, the medication status was not systematically controlled across all participants, and dopaminergic effects on gait and cognition could not be fully excluded. Fifth, the subgroup analyses comparing PDCN and PDMCI were exploratory in nature, and the relatively small sample sizes within each subgroup warrant caution when interpreting these findings. Sixth, secondary task performance data during the cell phone use condition, such as typing accuracy, completion rate, and response speed, were not recorded. The absence of such data limits our ability to determine whether the observed gait changes reflect genuine cognitive-motor interference, differences in task prioritization strategies, variability in smartphone familiarity across participants, or individual differences in subjective cognitive load. Future longitudinal studies with larger, well-characterized cohorts are needed to replicate the exploratory subgroup and moderation findings reported here, clarify the temporal relationships between gait deterioration and cognitive decline in PD, and further evaluate the clinical utility of dual-task gait assessment, particularly the cell phone use paradigm, as a sensitive marker of cognitive–motor decline in early PD. Future studies should also incorporate simultaneous monitoring of both gait and secondary task performance to enable more comprehensive interpretation of cognitive-motor dual-task findings.

## Data Availability

The raw data supporting the conclusions of this article will be made available by the authors, without undue reservation.

## References

[ref1] Ali P. Dinomais M. Labriffe M. Pieruccini-Faria F. Montero-Odasso M. Bartha R. . (2025). Mapping the neural substrate of high dual-task gait cost in older adults across the cognitive spectrum. Brain Struct. Funct. 230:25. doi: 10.1007/s00429-024-02873-6, 39755775 PMC11700055

[ref2] Amara A. W. Wood K. H. Miften A. M. Kleinschmidt L. White C. S. Joop A. . (2025). Gait and balance dysfunction are associated with cognitive performance only in men with Parkinson's disease. Clin. Park. Relat. Disord. 13:100363. doi: 10.1016/j.prdoa.2025.100363 40687115 PMC12274842

[ref3] Amboni M. Barone P. Hausdorff J. M. (2013). Cognitive contributions to gait and falls: evidence and implications. Mov. Disord. 28, 1520–1533. doi: 10.1002/mds.25674, 24132840 PMC4119872

[ref4] Amboni M. Ricciardi C. Cuoco S. Donisi L. Volzone A. Ricciardelli G. . (2022). Mild cognitive impairment subtypes are associated with peculiar gait patterns in Parkinson's disease. Front. Aging Neurosci. 14:781480. doi: 10.3389/fnagi.2022.781480, 35299943 PMC8923162

[ref5] Clark D. J. (2015). Automaticity of walking: functional significance, mechanisms, measurement and rehabilitation strategies. Front. Hum. Neurosci. 9:246. doi: 10.3389/fnhum.2015.00246, 25999838 PMC4419715

[ref6] De Baene W. Duyck W. Brass M. Carreiras M. (2015). Brain circuit for cognitive control is shared by task and language switching. J. Cogn. Neurosci. 27, 1752–1765. doi: 10.1162/jocn_a_00817 25901448

[ref7] Di Filippo F. De Biasi G. Russo M. Ricciardi C. Pisani N. Volzone A. . (2025). Dual-task-related gait patterns as possible marker of precocious and subclinical cognitive alterations in Parkinson disease. Sci. Rep. 15:3371. doi: 10.1038/s41598-025-85118-8, 39870679 PMC11772767

[ref8] Doi T. Makizako H. Shimada H. Park H. Tsutsumimoto K. Uemura K. . (2013). Brain activation during dual-task walking and executive function among older adults with mild cognitive impairment: a fNIRS study. Aging Clin. Exp. Res. 25, 539–544. doi: 10.1007/s40520-013-0119-5, 23949972

[ref9] Galna B. Lord S. Burn D. J. Rochester L. (2015). Progression of gait dysfunction in incident Parkinson's disease: impact of medication and phenotype. Mov. Disord. 30, 359–367. doi: 10.1002/mds.26110 25546558

[ref10] Goldman J. G. Weis H. Stebbins G. Bernard B. Goetz C. G. (2012). Clinical differences among mild cognitive impairment subtypes in Parkinson's disease. Mov. Disord. 27, 1129–1136. doi: 10.1002/mds.25062, 22778009 PMC3412930

[ref11] Gonzalez M. C. Dalen I. Maple-Grødem J. Tysnes O. B. Alves G. (2022). Parkinson's disease clinical milestones and mortality. NPJ Parkinsons Dis. 8:58. doi: 10.1038/s41531-022-00320-z 35550520 PMC9098431

[ref12] Hjelle N. Mohanty B. Hubbard T. Johnson M. D. Wang J. Johnson L. A. . (2025). Impairment of neuronal activity in the dorsolateral prefrontal cortex occurs early in parkinsonism. Front. Neurosci. 19:1521443. doi: 10.3389/fnins.2025.1521443, 39896336 PMC11782136

[ref13] Holtzer R. Mahoney J. R. Izzetoglu M. Izzetoglu K. Onaral B. Verghese J. (2011). FNIRS study of walking and walking while talking in young and old individuals. J. Gerontol. A Biol. Sci. Med. Sci. 66, 879–887. doi: 10.1093/gerona/glr068, 21593013 PMC3148759

[ref14] Huxhold O. Li S. C. Schmiedek F. Lindenberger U. (2006). Dual-tasking postural control: aging and the effects of cognitive demand in conjunction with focus of attention. Brain Res. Bull. 69, 294–305. doi: 10.1016/j.brainresbull.2006.01.002, 16564425

[ref15] Johansson H. Ekman U. Rennie L. Peterson D. S. Leavy B. Franzén E. (2021). Dual-task effects during a motor-cognitive task in Parkinson's disease: patterns of prioritization and the influence of cognitive status. Neurorehabil. Neural Repair 35, 356–366. doi: 10.1177/1545968321999053, 33719728 PMC8073879

[ref16] Kang Y. Park J. S. Yu K. H. Lee B. C. (2009). A reliability, validity, and normative study of the Korean-Montreal cognitive assessment (K-MoCA) as an instrument for screening of vascular cognitive impairment. Korean J. Clin. Psychol. 28, 549–562. doi: 10.15842/kjcp.2009.28.2.013

[ref17] Kelly V. E. Eusterbrock A. J. Shumway-Cook A. (2012). A review of dual-task walking deficits in people with Parkinson’s disease: motor and cognitive contributions, mechanisms, and clinical implications. Parkinsons Dis. 2012:918719. doi: 10.1155/2012/918719 22135764 PMC3205740

[ref18] Kusleikiene S. Pukenas K. Drozdova Statkeviciene M. Ziv G. Vints W. A. J. Masiulis N. . (2025). Relationship between balance automaticity and dual-task interference in older adults. Mot. Control. 30, 100–116. doi: 10.1123/mc.2025-0014, 41468227

[ref19] Lee Y. Shin S. (2024). Risk of using smartphones while walking for digital natives in realistic environments: effects of cognitive-motor interference. Heliyon 10:e28901. doi: 10.1016/j.heliyon.2024.e28901, 38601574 PMC11004577

[ref20] Litvan I. Goldman J. G. Tröster A. I. Schmand B. A. Weintraub D. Petersen R. C. . (2012). Diagnostic criteria for mild cognitive impairment in Parkinson's disease: Movement Disorder Society task force guidelines. Mov. Disord. 27, 349–356. doi: 10.1002/mds.24893, 22275317 PMC3641655

[ref21] Miyamoto M. Miyamoto T. (2022). Montreal cognitive assessment predicts the short-term risk of Lewy body disease in isolated REM sleep behavior disorder with reduced MIBG scintigraphy. Mov. Disord. Clin. Pract. 10, 32–41. doi: 10.1002/mdc3.13569, 36698993 PMC9847289

[ref22] Monaghan A. S. Ragothaman A. Harker G. R. Carlson-Kuhta P. Horak F. B. Peterson D. S. (2023). Freezing of gait in Parkinson's disease: implications for dual-task walking. J. Parkinsons Dis. 13, 1035–1046. doi: 10.3233/jpd-230063, 37574744 PMC10578213

[ref23] Msigwa S. S. Hong S. Mkwambe M. C. Marealle E. Zhang X. Wang J. Y. (2026). Dual-task gait cognitive-motor interference as a marker of cognitive impairment in Parkinson's disease: a systematic review and meta-analysis. Mov. Disord. Clin. Pract. 13, 876–888. doi: 10.1002/mdc3.70409, 41174907 PMC13071337

[ref24] Mukli P. Muranyi M. Lipecz Á. Szarvas Z. Csípő T. Ungvari A. . (2025). Age-related and dual task-induced gait alterations and asymmetry: optimizing the Semmelweis study gait assessment protocol. Geroscience 47, 6955–6983. doi: 10.1007/s11357-025-01722-6, 40478431 PMC12638531

[ref25] Nedović N. Eminović F. Marković V. Stanković I. Radovanović S. (2024). Gait characteristics during dual-task walking in elderly subjects of different ages. Brain Sci. 14:148. doi: 10.3390/brainsci14020148, 38391723 PMC10886897

[ref26] Nedović N. Mutavdžin Krneta S. Jovanović S. Vujičić D. Kozinc Ž. Skvortsov D. (2025). Dual task gait analysis: combined cognitive motor demands most severely impact walking patterns and joint kinematics. Life 15:1009. doi: 10.3390/life15071009, 40724512 PMC12300433

[ref27] Ohsugi H. Ohgi S. Shigemori K. Schneider E. B. (2013). Differences in dual-task performance and prefrontal cortex activation between younger and older adults. BMC Neurosci. 14:10. doi: 10.1186/1471-2202-14-10, 23327197 PMC3552708

[ref28] Patel P. Lamar M. Bhatt T. (2014). Effect of type of cognitive task and walking speed on cognitive-motor interference during dual-task walking. Neuroscience 260, 140–148. doi: 10.1016/j.neuroscience.2013.12.016, 24345478

[ref29] Postuma R. B. Berg D. Stern M. Poewe W. Olanow C. W. Oertel W. . (2015). MDS clinical diagnostic criteria for Parkinson's disease. Mov. Disord. 30, 1591–1601. doi: 10.1002/mds.26424, 26474316

[ref30] Riedel N. Herzog M. Stein T. Deml B. (2024). Cognitive-motor interference during walking with modified leg mechanics: a dual-task walking study. Front. Psychol. 15:1375029. doi: 10.3389/fpsyg.2024.1375029, 38699569 PMC11063364

[ref31] Tombu M. Jolicoeur P. (2003). A central capacity sharing model of dual-task performance. J. Exp. Psychol. Hum. Percept. Perform. 29, 3–18. doi: 10.1037/0096-1523.29.1.3, 12669744

[ref32] Verghese J. Robbins M. Holtzer R. Zimmerman M. Wang C. Xue X. . (2008). Gait dysfunction in mild cognitive impairment syndromes. J. Am. Geriatr. Soc. 56, 1244–1251. doi: 10.1111/j.1532-5415.2008.01758.x, 18482293 PMC2574944

[ref33] Yamada P. A. Amaral-Felipe K. M. Spinoso D. H. Abreu D. C. C. Stroppa-Marques A. E. Z. Faganello-Navega F. R. (2020). Everyday tasks impair spatiotemporal variables of gait in older adults with Parkinson's disease. Hum. Mov. Sci. 70:102591. doi: 10.1016/j.humov.2020.102591 32217209

[ref34] Yogev-Seligmann G. Hausdorff J. M. Giladi N. (2008). The role of executive function and attention in gait. Mov. Disord. 23, 329–342. doi: 10.1002/mds.21720, 18058946 PMC2535903

[ref35] Yogev-Seligmann G. Hausdorff J. M. Giladi N. (2012). Do we always prioritize balance when walking? Towards an integrated model of task prioritization. Mov. Disord. 27, 765–770. doi: 10.1002/mds.24963, 22419512

[ref36] Yoo D. Lee J. Y. Kim Y. K. Yoon E. J. Kim H. Kim R. . (2021). Mild cognitive impairment and abnormal brain metabolic expression in idiopathic REM sleep behavior disorder. Parkinsonism Relat. Disord. 90, 1–7. doi: 10.1016/j.parkreldis.2021.07.022, 34314988

[ref37] Zhang W. Ling Y. Chen Z. Ren K. Chen S. Huang P. . (2024). Wearable sensor-based quantitative gait analysis in Parkinson's disease patients with different motor subtypes. NPJ Digit. Med. 7:169. doi: 10.1038/s41746-024-01163-z, 38926552 PMC11208588

