# Supplementary material for: Dual-task gait and gait-cognition coupling in patients with Parkinson’s disease versus healthy older adults: moderation by cognitive status
Source: Front Aging Neurosci. 2026 Aug 12;18:1896308. doi: 10.3389/fnagi.2026.1896308 (PMC13506864; doi:10.3389/fnagi.2026.1896308)
Supplement: Supplementary file 1 [file Table_1.DOCX]

Supplementary Table 1. Neuropsychological test performance in patients with Parkinson’s disease stratified by cognitive status

| Domain | Test | PDCN (N=38) | PDMCI (N=27) | *p* value |
| --- | --- | --- | --- | --- |
| Attention | DST_FB | -0.060 ± 1.392 | -0.729 ± 1.203 | 0.090^1^ |
|  | CWST.CR | -0.008 ± 1.078 | -2.130 ± 2.763 | **<0.001^1^** |
| Language | K-BNT | 0.798 ± 0.795 | -0.232 ± 1.299 | **0.001^2^** |
| Visuospatial | RCFTscore | 0.156 ± 1.037 | -1.207 ± 1.804 | **<0.001^1^** |
| Memory | SVLT_IR | 0.443 ± 1.106 | -0.276 ± 0.872 | **<0.001^2^** |
|  | SVLT_DR | 0.028 ± 0.977 | -1.361 ± 1.375 | **<0.001^1^** |
|  | SVLT_Rec | 0.197 ± 0.862 | -0.344 ± 0.843 | **0.014^2^** |
|  | RCFT_IR | 0.188 ± 1.084 | -1.204 ± 0.955 | **<0.001^1^** |
|  | RCFT_DR | 0.253 ± 0.895 | -1.170 ± 1.007 | **<0.001^2^** |
|  | RCFT_Rec | 0.326 ± 1.054 | -0.543 ± 1.057 | **0.002^2^** |
| Executive | COWATsem | -0.206 ± 0.890 | -0.863 ± 1.099 | **0.010^2^** |
|  | COWATpho | 0.126 ± 0.846 | -1.094 ± 0.990 | **<0.001^2^** |

Values represent age-, sex-, and education-adjusted Z-scores and are presented as mean ± standard deviation. Group differences between PDCN and PDMCI were assessed using the Mann-Whitney U test or independent t-test, as appropriate. Significant differences (*p*<0.05) are shown in bold.

Abbreviations: BNT, Boston Naming Test; COWAT, Controlled Oral Word Association Test; CWST-CR, Color Word Stroop Test–Color Reading; DST-FB, Digit Span Test Forward/Backward; PDCN, Parkinson's disease with cognitively normal; PDMCI, Parkinson's disease with mild cognitive impairment; RCFT, Rey-Osterrieth Complex Figure Test; SVLT, Seoul Verbal Learning Test.
